# Searching for optimal blood pressure targets in type 2 diabetic patients with coronary artery disease

**DOI:** 10.1186/s12933-019-0959-1

**Published:** 2019-11-16

**Authors:** Ying Shen, Yang Dai, Xiao Qun Wang, Rui Yan Zhang, Lin Lu, Feng Hua Ding, Wei Feng Shen

**Affiliations:** 1 Department of Cardiology, Rui Jin Hospital, Shanghai Jiao Tong University School of Medicine, Shanghai, 200025 People’s Republic of China; 2 Institute of Cardiovascular Disease, Shanghai Jiao Tong University School of Medicine, Shanghai, 200025 People’s Republic of China

**Keywords:** Type 2 diabetes mellitus, Coronary artery disease, Hypertension, Blood pressure

## Abstract

**Background:**

Controversies exist regarding the optimal blood pressure (BP) level that is safe and provides cardiovascular protection in patients with type 2 diabetes mellitus (T2DM) and coexistent coronary artery disease. Several new glucose-lowering agents have been found to lower BP as well, making the interaction between BP and T2DM even more complex.

**Methods:**

With the reference to recent literature, this review article describes the potential mechanisms of increased risk of hypertension in T2DM and outlines the possible optimal BP levels based upon recommendations on the management of hypertension by the current guidelines, in combination with our research findings, for type 2 diabetic patients with coronary artery disease.

**Results:**

The development of hypertension in T2DM involves multiple processes, including enhanced sympathetic output, inappropriate activation of renin-angiotensin- aldosterone system, endothelial dysfunction induced through insulin resistance, and abnormal sodium handling by the kidney. Both AGE-RAGE axis and adipokine dysregulation activate intracellular signaling pathways, increase oxidative stress, and aggravate vascular inflammation. Pancreatic β-cell specific microRNAs are implicated in gene expression and diabetic complications. Non-pharmacological intervention with lifestyle changes improves BP control, and anti-hypertensive medications with ACEI/ARB, calcium antagonists, β-blockers, diuretics and new hypoglycemic agent SGLT2 inhibitors are effective to decrease mortality and prevent major adverse cardiovascular events. For hypertensive patients with T2DM and stable coronary artery disease, control of BP < 130/80 mmHg but not < 120/70 mmHg is reasonable, whereas for those with chronic total occlusion or acute coronary syndromes, an ideal BP target may be somewhat higher (< 140/90 mmHg). Caution is advised with aggressive lowering of diastolic BP to a critical threshold (< 60 mmHg).

**Conclusions:**

Hypertension and T2DM share certain similar aspects of pathophysiology, and BP control should be individualized to minimize adverse events and maximize benefits especially for patients with T2DM and coronary artery disease.

## Background

Epidemiologic studies have shown that hypertension and type 2 diabetes mellitus (T2DM) are global public health issues and become the major cause of disease burden and mortality [1, 2]. The World Health Organization estimated that 40% of adults worldwide have hypertension (about 90% are classified with essential hypertension) [3], and approximately 422 million adults were living with diabetes(more than 90% are T2DM) [4]. In addition, hypertension is present in more than half of type 2 diabetic patients and contributes significantly to macro- and micro-vascular complications [5]. The development of T2DM is often asymptomatic and subclinical for a long period, and before diagnosis of T2DM, individuals can reside in the high-risk state of prediabetes, defined as impaired fasting glucose or impaired glucose tolerance [6, 7]. Recently, the prevalence of hypertension and T2DM is increasing in many Asian countries, with a number of countries with blood pressure (BP) and glucose above the global average [7–12]. The Chinese National Report of Cardiovascular Disease 2018 pointed out that the prevalence of hypertension and diabetes reaches 23.2% and 10.9%, respectively, leading to an estimate of about 290 million of adult people suffering from cardiovascular disease in China [9]. The major goal for cardiovascular care is to prevent morbidity and mortality by controlling glucose, normalizing BP, and reducing other cardiovascular risk factors. Data frequently suggest an existence of the relationship between BP and cardiovascular risks as low as 110–115 mmHg for systolic BP and 70–75 mmHg for diastolic BP. Every 20 mmHg systolic and 10 mmHg diastolic BP increase above the threshold has shown to double the risk of mortality from ischemic heart disease and stroke [10]. For decades, clinical practice guidelines vary in determining the optimal BP target in patients with T2DM. Whereas several guidelines recommend a BP goal of < 140/90 mmHg [13, 14], some recommend a lower target of systolic and diastolic BP in certain diabetic population [15, 16]. The newly released American College of Cardiology (ACC)/American Heart Association (AHA) Guideline for the Prevention, Detection, Evaluation, and Management of High BP in adults supports a more aggressive diagnostic and treatment approach, recommending hypertensive patients to maintain their BP < 130/80 mmHg [17]. Although the adoption of new guideline is expected to increase the prevalence of hypertension, endorsing the aggressive approach including lifestyle change and medical treatment would lead to reduced risk of major adverse cardiac events and improvement in overall clinical outcome [10, 17]. However, controversies exist regarding the optimal level of BP attained with therapeutic interventions that is safe and provides cardiovascular protection, especially in patients with T2DM and coexistent coronary artery disease [18, 19]. Furthermore, the class of drugs most appropriate for the treatment of hypertensive diabetics is also unclear and different guidelines emphasize use of different classes for anti-hypertensive treatment in type 2 diabetic patients [16]. Particularly, several new glucose-lowering agents for the treatment of diabetes have been found to lower BP as well, making the interaction between BP and T2DM even more complex [20]. In this review, we will outline the possible optimal BP levels based upon recommendations on the management of hypertension by the current guidelines, in combination with our research findings, for type 2 diabetic patients with coronary artery disease.

## Mechanisms of increased risk of hypertension in type 2 diabetes

**Fig. 1:**
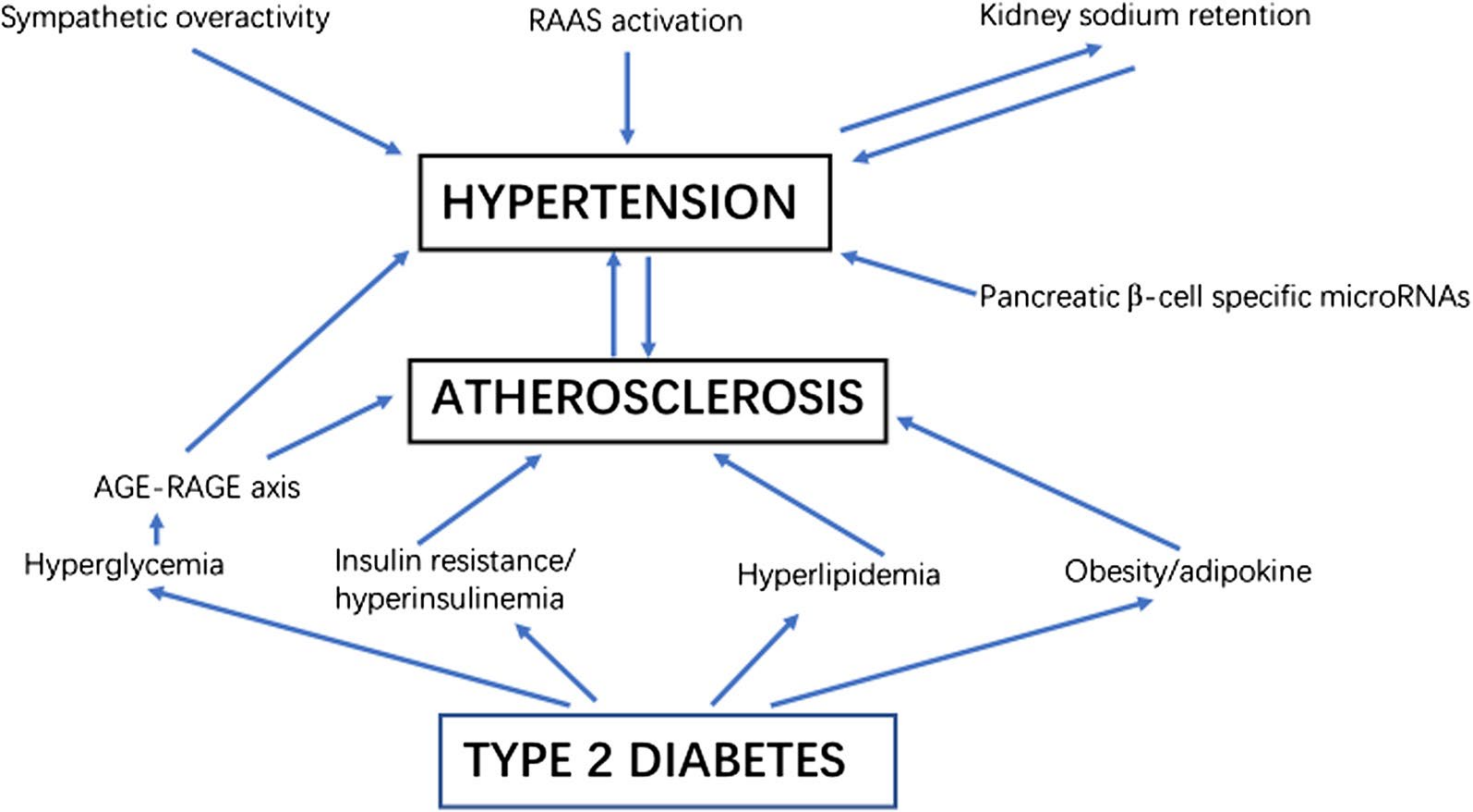
Mechanism of hypertension and coronary atherosclerosis in type 2 diabetes

Obviously, the actual mechanism of hypertension in T2DM is complex and multi-factorial,including enhanced sympathetic output, inappropriate activation of the renin-angiotensin-aldosterone system (RAAS), oxidative stress, inflammation, insulin resistance-mediated endothelial dysfunction, and abnormal sodium handling by the kidney (Fig. 1). Nevertheless, both T2DM and hypertension share certain similar aspects of pathophysiology [21, 22].

### Insulin resistance/hyperinsulinemia

Approximately 50% of hypertensive patients manifest systemic insulin resistance or hyperinsulinemia, which plays an important role in the development of both T2DM and hypertension [23]. Loss of sensitivity to insulin action principally affects glucose and lipid metabolism, e.g., sparing insulin’s action to retain sodium in the distal tubule [16, 24]. When insulin-mediated glucose uptake is reduced, the secretion of insulin is increased to maintain homeostasis. Insulin resistance or hyperinsulinemia is frequently associated with a low-grade inflammation of endothelial and smooth muscle cells in the vascular wall, which induces endothelial dysfunction, vascular stiffness, hypertrophy, fibrosis and remodeling [25]. It is also found that insulin resistance or hyperinsulinemia enhances sympathetic output and disrupts the intricate physiological balance in vascular tone and vessel growth, leading to reduced arterial compliance, a characteristic phenotype in hypertension [26]. Abundant evidence suggests that impaired endothelium-dependent vasodilation may in turn contribute to or exacerbate insulin resistance by limiting the delivery of substrate (glucose) to key target tissue [21–25].

### Obesity/adipokines

Obesity (particularly increased visceral adiposity) is a key pathogenic factor behind the coexistence of both T2DM and hypertension [27]. There is increasing evidence that increased afferent traffic from and efferent activity to the kidney promotes the development of hypertension associated with obesity and insulin resistance [23]. Chronic low-grade inflammation and oxidative stress in the adipose tissue contributes to systemic elevation in BP, in part, through local production of components of the RAAS. Activation of angiotensin II type 1 receptor in non-adrenal tissues causes multiple intracellular events, including production of reactive oxygen species (ROS), reduced insulin metabolic signaling, and increased proliferative and inflammatory vascular responses [28]. Adipose tissue is known to produce a lipid-soluble factor that stimulates aldosterone production from the adrenal zona glomerulosa [29, 30]. Aldosterone activation of the mineralocorticoid receptor in the renal distal tubule and collecting duct increases sodium retention, leading to expansion of plasma volume and increased BP [31]. In addition, aldosterone exerts non-genomic actions likely via mineralocorticoid receptor activation, which contribute to hypertension by altering cellular redox state, signaling and endothelial-mediated vascular relaxation [30, 31].

Dysregulation of adipose tissue-derived adipokines is involved in the development of proliferative and inflammatory vascular diseases, including hypertension. Adiponectin, a protein widely implicated in the pathogenesis of insulin resistance, has a profound effect on metabolism and vasculature and conveys anti-hypertensive properties [32, 33]. In several population-based studies, levels of circulating adiponectin have been shown to be inversely proportional to adiposity (body mass index) and burden of hypertension and T2DM [34]. Mechanisms underlying BP lowering effects of adiponectin remains unclear but expression of vascular endothelial nitric oxide (NO) synthase (eNOS) and prostaglandin I_2_ synthase may play a role [35]. Adiponectin has been suggested to have sympatho-inhibitory action and may also protect against incident hypertension through its anti-inflammatory effects [36]. Leptin, an adipokine elevated in obese individuals, increases sympathetic output likely through a central nervous system effect involving leptin receptor activation [26]. The C1q/TNF-related protein 1 (CTRP1) is expressed at high levels in adipose tissue by proinflammatory cytokines, and increased levels of CTRP1 are associated with the extent of coronary atherosclerosis [37] and reduced collateral formation in patients with chronic coronary total occlusion (CTO) [38, 39]. It was also revealed that CTRP1 is expressed in glomerulosa of the adrenal cortex and stimulates production of aldosterone, suggesting that angiotensin II-induced aldosterone production is, at least in part, mediated by the stimulation of CTRP1 secretion [40]. In addition, circulating levels of CTRP1 were significantly up-regulated in obese subjects as well as hypertensive patients [40–42]. Taken together, these observations support a notion that CTRP1 may be a newly identified molecular link between obesity and hypertension.

### AGEs-RAGE axis

Chronic hyperglycemia and altered redox state in T2DM increase the formation and accumulation of advanced glycation end-products (AGE). Binding of AGE to receptor for AGE (RAGE) triggers several intracellular signaling pathways and increases the expression and release of inflammatory cytokines, generation of ROS, and activates nuclear factor kappa-B. These alterations might produce contraction of the arterial wall [43], which could reduce arterial pliability and increase vascular stiffness, particularly leading to a rise in the systolic BP and a widening of pulse pressure [44]. In patients with hypertension, there is a positive correlation between plasma levels of AGE and arterial stiffness, and an inverse association between arterial stiffness and serum levels of soluble RAGE (sRAGE) and endogenous secretory RAGE (esRAGE) [43]. The properties of collagen and elastin are altered through AGE–RAGE intermolecular covalent bond or cross-linking [45], which make them less susceptible to hydrolytic turn-over and more reduced elasticity of the arterial wall. Besides structural changes in the artery (increased collagen and decreased elastin), interaction of AGE with RAGE may also induce hypertension through production of ROS, such as superoxide anion, hydrogen peroxide and hydroxyl radicals irrespective of arterial stiffness [43]. Diabetic dyslipidemia (elevated concentration of small dense low-density lipoprotein cholesterol, high concentration of triglycerides, triglyceride-rich remnants, very low-density lipoprotein cholesterol and apoprotein B, usually in combination with low levels of high-density lipoprotein [HDL] cholesterol) will cause endovascular toxicity. Previous studies have shown that the clearance of AGE-modified low-density lipoprotein was reduced in patients with T2DM, causing a significant increase in oxide low-density lipoprotein (oxLDL) [46, 47]. At the same time, HDL can also be glycated, which decrease its ability of reverse transportation of cholesterol and increase the formation of foam cells after enhanced uptake of oxLDL by mononuclear cells. We found that glycated HDL decreases the activity of paraoxonase (PON) [48]. Patients with T2DM and coronary artery disease had elevated plasma levels of glycated apoprotein A-I and A-IV and decreased PON-1 and -3. These biochemical changes are strongly associated with the severity and progression of coronary artery disease [48–50] and reduced coronary collateralization [51].

### Diabetic nephropathy

It is well recognized that kidney and cardiovascular system are inextricably interlinked as determinants of ambient BP levels in both normal and diseased conditions. The complex interplay between renal disease and hypertension appears to be especially evident in patients with T2DM, who are inherently at high risk for progressive glomerular damage via vascular fibrosis, calcification, prothrombotic effects, and vascular damage [22]. It has been reported that almost 40% of patients with T2DM (especially for elderly) are already hypertensive at diagnosis, and nephropathy is one of the major microvascular complications of T2DM. In fact, approximately 85% of patients with overt diabetic nephropathy have hypertension [21]. In a bi-directional manner, the incidence and severity of hypertension increases with the emergence and progression of nephropathy.

### MicroRNA

Recent studies showed that a number of pancreatic β-cell specific microRNAs, a group of noncoding RNAs that are multifunctional, are implicated in the gene expression and various disease processes. For example, diabetic vascular complications are associated with increased levels of miR-223, miR-320, miR-501, miR-504 and miR-1 and decreased levels of miR-16, miR-133, miR-492 and miR-373 [5, 52].

## BP levels in diabetic patients with stable coronary artery disease

The beneficial effects of anti-hypertensive drugs on clinical and cardiovascular outcomes are well established [53], and strict BP control is strongly recommended by most previous guidelines for general patients [13–15, 17]. However, this therapeutic strategy has been challenged in hypertensive patients with T2DM [16, 18, 19], especially for those with coronary artery disease [54, 55].

### Systolic BP

Data from several landmark trials and meta-analyses demonstrate benefit of decreased systolic BP with intensive BP control in reducing the risk of ischemic as well as hemorrhagic stroke for patients with T2DM and hypertension [56–58]. Recently, the SPRINT (Systolic Blood Pressure Intervention Trial) reported that for non-diabetic patients with increased cardiovascular risk, intensive BP control (target systolic BP < 120 mmHg) was associated with 25% lower rate of primary composite outcome after 3.26 years of follow-up compared with standard BP goal (target systolic BP < 140 mmHg) [59]. However, these beneficial effects of intensive BP lowering seen in non-diabetic patients have not been demonstrated in patients with T2DM [54, 60–64]. In fact, the results of prospective ACCORD (Action to Control Cardiovascular Risk in Diabetes) trial showed no differences in composite outcome of cardiovascular death, non-fatal myocardial infarction and non-fatal stroke between intensive and standard BP control [60, 61]. An observational analysis of the INVEST (International Verapamil SR-Trandolapril) study revealed that all-cause mortality was increased in diabetic patients with systolic BP < 115 mmHg [62]. A subgroup analysis of the INVEST study involving 6400 patients who were at least 50 years old and had diabetes and coexistent coronary artery disease showed that tight control of systolic BP was not associated with improved cardiovascular outcomes compared with usual BP control [63]. In an international, prospective, longitudinal registry including 22,672 patients with stable coronary artery disease and treated for hypertension, systolic and diastolic BP before each event were averaged and categorized into 10 mmHg increments. After a median follow-up of 5 years, systolic BP > 140 mmHg or < 120 mmHg was correlated with increased risks of cardiovascular mortality, myocardial infarction, or stroke [54]. Recently, Bohm et al. reported the results of the secondary analyses of ONTARGET (Ongoing Telmisartan Alone and in combination with Ramipril Global Endpoint Trial) and TRANSCEND (Telmisartan Randomized Assessment Study in ACE Intolerant Subjects with CV Disease). These trials aimed to assess the risk in patients with and without diabetes over the whole spectrum of achieved systolic and diastolic BP. The results have shown that mean achieved in-trial systolic BP < 120 mmHg was associated with 1.53-fold increased risk for combined outcome in patients with diabetes [64]. The overall findings thereby underscore the need for caution when aggressive lowering BP therapy is applied, and further question the concept of ‘lower BP is better’ for hypertensive patients with T2DM and coronary artery disease.

### Diastolic BP

Since physiological coronary blood flow predominantly occurs during diastole, diastolic BP would be expected to have greater clinical relevance. The INVEST study showed that cardiovascular risk was reduced for type 2 diabetic patients with a diastolic BP < 90 mmHg but was increased for those with a diastolic BP < 70 mmHg [62]. Similarly, the results of the secondary analyses of ONTARGET and TRANSCEND trials also showed that a diastolic BP < 70 mmHg was associated with increased risk for the combined outcome in diabetic and non-diabetic patients, and also for all other endpoints except stroke [64]. These data suggest that cardiovascular risk may be defined by diastolic BP levels, despite optimally achieved systolic BP. In patients with hypertension, the Framingham Heart Study also showed that the same cutoff point of diastolic BP was associated with increased cardiovascular events. Furthermore, the risk was increased among those with both low diastolic BP and a wide pulse pressure [65].

The importance of optimal diastolic BP levels in determining clinical outcomes for patients with coronary artery disease was further substantiated by several recent studies. Peri-Okonny et al. assessed the relationship between reduced diastolic BP and occurrence of angina in a cohort of 1259 patients with stable coronary artery disease (more than one-third of them had diabetes). In the unadjusted model, diastolic BP was associated with angina with a J-shaped relationship (p for nonlinearity = 0.027), with a progressive increase in odds of angina as diastolic BP below 70–80 mmHg. Patients with a diastolic BP of 60 mmHg had 1.37-fold increased risk of angina compared with those having a diastolic BP of 80 mmHg. This association remained significant after adjustment for demographics, comorbidities, heart rate, systolic BP, and anti-angina and anti-hypertensive medications [66].

Angiographically-documented CTO occurs in around 20–30% of type 2 diabetic patients with or without hypertension, especially for those with multi-vessel disease [67]. Percutaneous coronary intervention(PCI)of chronic totally occluded lesions with drug-eluting stent implantation as a part of complete revascularization has become a routine clinical practice [68]. We classified 431 type 2 diabetic and 287 non-diabetic patients with stable angina and angiographic total occlusion of at least one major coronary artery according to 10 mmHg increments of diastolic BP from < 60 to ≥ 100 mmHg and systolic BP from < 100 to ≥ 180 mmHg. The results showed that diastolic BP was related to the degree of coronary collateral formation in a U-shaped pattern, with the lowest risk of poor collateralization at diastolic BP 80–89 mmHg for patients with T2DM and at 90–99 mmHg for non-diabetic counterparts, respectively [69]. In an additional study, we assessed the interactive effects of predominant collateral donor artery (PCDA) stenosis and BP on coronary collateral flow to the chronically occluded bed in 200 type 2 diabetic patients and 200 age- and sex- matched non-diabetic controls. Collateral flow index (CFI) was determined by simultaneous recording of central aortic pressure and intracoronary pressure distal to the occluded segment during PCI. The study demonstrated that when the PCDA was mildly stenotic, CFI was gradually increased along with a reduction in aortic diastolic BP, but it was decreased when diastolic BP was below 60 mmHg in type 2 diabetic patients, with a relative reduction of 32.1% compared with non-diabetic controls. In the presence of moderate PCDA stenosis, with decreasing diastolic BP, the difference of CFI between type 2 diabetic patients and non-diabetic controls was gradually increased. When diastolic BP was below 80 mmHg, patients with T2DM had a significantly lower CFI compared to non-diabetic controls, with a relative reduction of 19.8% at diastolic BP 70–79 mmHg, 28.2% at 60–69 mmHg and 38.2% below 60 mmHg, respectively. A severe stenotic lesion in the PCDA always led to a more pronounced decrease in CFI, with a relative reduction of 37.3% for type 2 diabetic patients compared to non-diabetic controls when diastolic BP was below 60 mmHg. Thus, presence of PCDA stenosis confers greater risk for reduced coronary collateral flow when diastolic BP is decreased. For patients with T2DM, even a moderate stenosis in the PCDA is associated with more pronounced collateral flow reduction as diastolic BP decreases below 80 mmHg compared with non-diabetic patients [70]. These different effects of the severity of PCDA stenosis on collateral flow relative to BP between type 2 diabetic and non-diabetic patients remains unclear, but a likely explanation is the presence of more diffuse coronary atherosclerosis and microvascular disease and various influence of coronary vascular tone in patients with T2DM [71, 72]. Nevertheless, these observations are consistent with the J-curve phenomenon relating the overly reduced or elevated diastolic BP to adverse outcomes [73], and substantiate the concept that coronary autoregulation may be exhausted with low diastolic BP in the setting of atherosclerotic narrowing of the epicardial coronary arteries.

## BP levels in diabetic patients with acute coronary syndrome

Recently, White et al. evaluated the relationships between achieved clinician-measured BP and cardiovascular outcomes in 5380 patients with T2DM and recent acute coronary syndromes of the EXAMINE (Examination of Cardiovascular Outcomes With Alogliptin Versus Standard of Care) trial. Risks of major adverse cardiac events and cardiovascular death or heart failure were analyzed using a Cox proportional hazard model with adjustment for baseline covariates in 10 mmHg increments of diastolic BP from ≤ 60 to > 100 mmHg and systolic BP from ≤ 100 to > 160 mmHg during 2-year follow-up. Systolic BP of 131 to 140 mmHg and diastolic BP of 81 to 90 mmHg were used as reference groups. They observed a U-shaped relationship between cardiovascular outcome and BP. Importantly, average follow-up BP < 130/80 mmHg was associated with worsened cardiovascular outcomes, and the degree of risk was notably greater for those who had achieved average follow-up BP of < 120/70 mmHg [19].

## BP management for type 2 diabetic patients with coronary artery disease

The anti-hypertensive strategies most appropriate to type 2 diabetic patients with coronary artery disease have been widely studied.

### General considerations

It becomes increasingly important to individualize BP treatment to minimize adverse events and maximize benefits. Non-pharmacological modalities include weight loss, increased potassium-based diet, reduced total intake of sodium and fat (especially saturated fat), and regular physical activity and exercise. Although the cardiovascular benefits of lifestyle changes were not evaluated in type 2 diabetic patients, their implementation seems reasonable as these measures could favorably affect glycemia, lipid profile and BP level [74]. Certainly, pharmacological therapy is effective to decrease mortality, prevent major adverse cardiovascular events including non-fatal myocardial infarction, stroke and heart failure, and slow the progression of pre-existing kidney disease in patients with T2DM. Based on available evidence, type 2 diabetic patients with persistent BP > 140/90 mmHg should be started on anti-hypertensive drug therapy [13, 14]. Notably, the anti-hypertensive strategy (including the choice of BP lowering agents) for type 2 diabetic patients with coronary artery disease should be individualized according to the clinical conditions of the patients. It is important to keep in mind that the degree of BP reduction per se is the major determinant of reduction in cardiovascular risk, superseding the choice of anti-hypertensive drugs; a dictum that is valid in patients with T2DM and coronary artery disease. Monotherapy with angiotensin-converting enzyme inhibitor (ACEI) or angiotensin receptor blocker (ARB) can attain BP target in certain type 2 diabetic patients with coronary artery disease, especially when BP is only modestly elevated. However, combination therapy is eventually required in many individuals with T2DM and coronary artery disease, and most guidelines recommend adding a calcium antagonist or diuretic to RAAS inhibitors as add-on therapy [4, 6, 8, 13–15]. The superiority of a calcium channel blocker over a thiazide as an addition to ACEI or ARB was shown in terms of reduction of cardiovascular events, renal protection and improvement in insulin resistance [75]. In obese patients or when volume overload is present, diuretics may be used as well, and sometimes the escalation of double-drug treatment to triple-drug therapy is required to improve BP control in type 2 diabetic patients with hypertension [16]. Fixed-dose combinations in a single pill may increase compliance compared with corresponding free-drug components given separately, as it simplifies treatment and thereby can improve adherence on the part of the patients [76].

Not so infrequently, elderly patients with T2DM with or without coronary artery disease may experience a high systolic BP in the presence of a low diastolic BP, reflecting increased aortic stiffness. In this circumstance, the lowering of systolic BP (< 140 mmHg) is clearly beneficial even at the price of further lowering diastolic BP. However, for patients with coronary artery disease and diastolic BP below 60 mmHg, caution is advised during treatment. Alternative medications for angina (e.g., ivabradine or isosorbide) and revascularization or other non-pharmacological interventions may be more beneficial as opposed to further titration of anti-hypertensive medications.

### Anti-hypertensive medications

Several classes of anti-hypertensive agents have been used in the treatment of patients with T2DM and coronary artery disease.

#### RAAS inhibitors

ACEI/ARB are the first-line anti-hypertensive drugs in type 2 diabetic patients with coronary artery disease because they have at least similar [77] or even greater cardiovascular protection and more effectively reduce risk of mortality, myocardial infarction, heart failure and stroke than other anti-hypertensive agents, particularly for high-risk patients [78, 79]. In addition, available literature demonstrates that blockade of the RAAS also has potential benefits beyond BP lowering effects, including renal protection, improvements in insulin resistance, inflammation, oxidative stress, and endothelial function and decrease in activation of matrix metalloproteinases, along with amelioration of vascular function and ventricular remodeling [80]. However, combined use of both ACEI and ARB does not yield additional benefits and is, in fact, not recommended. Aldosterone antagonists such as spironolactone or eplerenone may be considered in type 2 diabetic patients with resistant hypertension as long as careful monitoring of renal function and serum potassium is made [81].

#### Calcium channel blockers

Calcium antagonists are commonly used for treating hypertension in type 2 diabetic patients with or without coronary artery disease, particularly in the elderly with isolated systolic hypertension [82]. In type 2 diabetic patients who require more than one drug for BP control, a combination of an ACEI or ARB and a dihydropyridine calcium channel blocker (such as amlodipine) is appropriate [83]. Calcium channel blockers were superior to thiazide diuretics in reducing cardiovascular events, with no disadvantages of worsening lipid and glucose uptake.

#### Diuretics

Despite some concern about the increased risk for metabolic and electrolytic disturbance, diuretics are effective for the treatment of hypertension in type 2 diabetic patients. In post hoc analyses of patients with hypertension and T2DM, thiazide resulted in a significant reduction in cardiovascular events, all-cause mortality, and hospitalization for heart failure compared to placebo, and generally was shown to be non-inferior to other antihypertensive agents. Benefits attributed to thiazide diuretics in terms of cardiovascular event reduction outweigh the risk of worsening glucose control in type 2 diabetic patients [84]. Low dose thiazides in combination with ACEI/ARB may minimize or prevent some of metabolic and electrolytic disturbance associated with diuretic therapy. Thiazide-like diuretics chlortalidone and indapamide were found to be less markedly associated metabolic abnormalities than hydrochlorothiazide but were as good as amlodipine or lisinopril in preventing fatal or non-fatal coronary artery disease and was more effective in hypertensive patients with T2DM [85]. However, the risk of worsening glucose control in type 2 diabetes and of new-onset diabetes in non-diabetic patients correlate with thiazide treatment due to its potential to negatively influence insulin resistance [86]. Thus, glucose and electrolytes should be monitored when initiating therapy.

#### β-adrenergic blockers

Hypertension is underpinned by high sympathetic nerve activity especially in younger or middle-age subjects. β-blockers reduce heart rate, decrease catecholamine-induced inflammatory reaction, and improve endothelial shear stress,which exert a beneficial effect on coronary blood flow and clinical outcomes [87]. β-blockers are frequently used as add-on treatment in hypertensive patients with coronary artery disease, heart failure or atrial fibrillation with rapid ventricular response [13]. Caution should be made when they were used in patients with T2DM due to its potential adverse metabolic effects, including an increase in triglyceride levels, a decrease in HDL cholesterol levels, weight gain, masking the worsening symptoms of hypoglycemia and aggravating insulin resistance [88]. Cavedilol with combined non-selective β and ɑ1 adrenergic antagonist actions improves survival in patients with heart failure and could not be as deleterious for glucose control [89].

### New hypoglycemic agents

The role of certain new anti-diabetic medications including dipeptidyl peptidase 4 (DPP-4) inhibitors, glucagon like peptide 1 (GLP-1)agonists, and sodium-glucose cotransporter 2 (SGLT 2) inhibitors, in BP control besides their glucose lowering effects in diabetic individuals has been investigated.

#### DPP-4 inhibitors and GLP-1 agonists

DPP-4 inhibitors increase endogenous GLP-1 by inhibiting the endogenous substance responsible for its degradation [16]. Several studies assessing the effect of treatment with DPP-4 inhibitors yielded conflicting results in terms of BP changes, with some showing a modest decrease in BP [90] and others revealing an increase in BP [91] or a counteraction against the hypotensive effects of ACEI [92]. GLP-1 agonists produce a mild BP reduction in clinical trials using office BP measurements [93, 94] but have no BP-lowering effect when using 24-h ambulatory BP monitoring [95]. On the other hand, GLP-1 agonists have been reported to increase heart rate via activation of the sympathetic nervous system [96]. Overall, both DPP-4 inhibitors and GLP-1 agonists appear to exert a neutral effect on BP and thus should not serve as an alternative to anti-hypertensive treatment in type 2 diabetic patients [16].

The CAROLINA (Cardiovascular Outcome Study of Linagliptin vs. Glimepiride in Type 2 Diabetes) randomized clinical trial examined the effect of treatment with the DPP-4 inhibitor linagliptin vs. the commonly used sulfonylurea glimepiride on cardiovascular safety in patients with relatively early T2DM and cardiovascular risk factors or established atherosclerotic cardiovascular disease. The results showed that the use of linagliptin compared with glimepiride over a median 6.3 years led to a noninferior risk of composite cardiovascular outcome [97]. Likewise, studies of the effects of GLP-1 agonists on clinical outcome have shown mixed results [98–100]. Although the EXSCEL (Exenatide Study of Cardiovascular Events Lowering) study has reported a potential cardiovascular benefit of once-weekly extended-release exenatide, the primary endpoint did not reach statistical significance [101]. These observations suggest that DPP-4 inhibitors and GLP-1 agonists may not improve clinical outcome for patients with T2DM.

#### SGLT2 inhibitors

These agents belong to a new class of unique oral glucose-lowering drugs and at the same time pertain multifaceted effects on hemodynamic and metabolic parameters beyond glycemic control [102, 103]. Recently, Mazidi et al. undertook a systematic review and meta-analysis of 43 randomized control trials (dapagliflozin: 22 trials; canagliflozin: 14 trials; empagliflozin: 4 trials; remogliflozin: 2 trials; pragliflozin: 1 trial) to determine the effect of SGLT2 inhibitors on BP among individuals with T2DM [104]. They found that the pooled estimate of the effect of SGLT2 on systolic BP levels was − 2.46 mmHg across all studies, − 2.23 mmHg across studies using canagliflozin, − 1.03 mmHg across studies using dapagliflozin, and − 2.59 mmHg across studies using empagliflozin. Likewise, the pooled estimate of the effect of SGLT2 inhibitors on diastolic BP levels was − 1.46 mmHg across all studies, − 2.23 mmHg across studies using canagliflozin, − 1.09 mmHg across studies using dapagliflozin, and − 2.59 mmHg across studies using empagliflozin. Further analyses revealed that the effect of SGLT2 inhibitors on systolic and diastolic BP was not influenced by length of follow-up, and remained similar across all studies and their subgroups [104]. These findings are consistent with previous reports and indicate that treatment with SGLT2 inhibitors either as monotherapy or add-on therapy with other drugs (such as ACEI/ARB), is associated with a small but significant reduction in systolic and diastolic BP measured in-office as well as by 24-h ambulatory BP monitoring, without increase in orthostatic hypotension [105–107].

The use of SGLT2 inhibitors also impacts favorably clinical outcome especially in patients with T2DM and cardiovascular disease [108]. In the EMPA-REG OUTCOME (Empagliflozin Cardiovascular Outcome Event Trial in Type 2 Diabetes Mellitus Patients) trial and the CANVAS (CANagliflozin cardioVascular Assessment Study) program, there was a reduction in primary composite cardiovascular endpoints with empagliflozin and canagliflozin, respectively, in high-risk patients with T2DM [109, 110]. The results of real-world observational CVD-REAL (Comparative Effectiveness of Cardiovascular Outcomes in New Users of Sodium-Glucose Cotransporter-2 Inhibitors) study support the cardiovascular benefits seen in the randomized trials [111]. Compared with DPP-4 inhibitors, some SGLT2 inhibitors have shown greater glycosylated hemoglobin (HbA1c) lowering in individuals with high baseline HbA1c levels [103, 112], greater weight loss, as well as greater BP lowering and satisfaction of renal function [103, 109, 110, 113]. Likewise, these agents may be also superior to DPP-4 inhibitors in terms of cardiovascular protection [114]. A pooled analysis of data from empagliflozin and canagliflozin trials supports a direct renal effect of SGLT2 inhibitors, including those receiving concomitant RAAS blockers [115, 116]. Notably, the risk of dehydration and urinary tract and genital infection is higher with SGLT2 inhibitors [109, 117].

The mechanism underlying the BP decrease by SGLT2 inhibitors remains unclear, and may include diuresis due to their chronic natriuretic and osmotic diuretic effects, weight loss, nephron remodeling, decrease in sympathetic overactivity and arterial stiffness, and increase in HDL cholesterol levels [118–120]. Therefore, SGLT2 inhibitors may play a significant role in reducing cardiovascular risk factors in people with T2DM. Canagliflozin and dapagliflozin inhibit SGLT2 activity in the proximal tubule, blocking the reabsorption of glucose back into the bloodstream. Furthermore, canagliflozin also blocks intestinal SGLT1, thereby reducing glucose absorption [118].

### BP control during PCI for CTO in type 2 diabetic patients

It is generally accepted that coronary revascularization with either drug-eluting stent-based PCI or coronary artery bypass grafting confers a substantial benefit to long-term outcome [67, 121]. In type 2 diabetic patients with multivessel disease especially at the presence of CTO, hypotension (especially low diastolic BP) should be avoided during PCI procedures, which could exacerbate myocardial ischemia. Similarly, if the vasodilatory reserve of the arterioles in the vascular bed supplied by a chronically occluded coronary artery is completely exhausted, whereas that of the PCDA is still preserved, coronary (collateral) steal may result. This phenomenon has been reported to occur in a very high proportion of well collateralized myocardial beds [122] and is most likely to occur in patients with moderate or severe stenosis of the PCDA, as vasodilator-induced increase in flow could cause a pressure drop across the stenotic lesions, thereby lowering collateral perfusion. Overall, multiple aspects should be taken into consideration when planning PCI procedure on patients with multi-vessel disease, including characteristics of totally occluded lesion, severity of PCDA stenosis, quality of collaterals, and clinical status of patients (diabetes and BP level). In type 2 diabetic patients with moderate PDCA stenosis, the use of fractional flow reserve to reveal ischemia may help in clinical decision-making [123], and warrants further investigation.

## Clinical perspective

The available literature and results of recent clinical studies and meta-analyses suggest that the primary BP goal in patients with established coronary artery disease is below 140/90 mmHg. Control of BP < 130/80 mmHg but not < 120/70 mmHg is reasonable for hypertensive and type 2 diabetic patients with stable coronary artery disease, whereas for those with CTO or acute coronary syndromes, an ideal BP target may be somewhat higher (< 140/90 mmHg) as recommended by the current guidelines [4, 13–15, 17]. Caution is advised with aggressive lowering of diastolic BP to a critical threshold (< 60 mmHg) which may result in no benefits but rather harmful particularly for hypertensive patients with T2DM and coronary artery disease [66, 124]. Likewise, in type 2 diabetic patients undergoing PCI for CTO, any excessive decrease in BP (especially low diastolic BP) before restoring anterograde flow of a chronic totally occluded lesion should be avoided during the procedure, because it may compromise collateral perfusion and exacerbate ischemia in the presence of at least moderate stenosis of the PCDA [69, 70]. In contrast, lower BP targets might be appropriate in those patients at higher risk of stroke and other micro-vascular complications such as chronic kidney disease, but this issue requires further study [125].

Most previous studies concerning the BP management of type 2 diabetic patients with coronary artery disease rely heavily on in-office BP measurement, however, ambulatory BP monitoring certainly improves baseline BP assessment and risk stratification after anti-hypertensive treatment [57, 126, 127]. Recent guidelines strongly recommended the use of ambulatory BP recording for accurate diagnosis of hypertension and individualized BP target in the treatment of patients with hypertension [13–15, 17]. Notably, concurrent masked hypertension and blunt response to nocturnal hypotension are not uncommon in patients with T2DM, which increases the risk of cardiovascular disease [128–130]. A large body of clinical evidence supports that BP lowering reduces macro- and micro-vascular complications in patients with T2DM. In future, it remains important to educate type 2 diabetic patients with coronary artery disease for increasing their treatment compliance and guideline adherence and improving the control rate of BP goal [131]. In addition, observational studies have demonstrated that there is often poor control of other cardiovascular risk factors in patients with T2DM [132]. Thus, to achieve the greatest risk reduction for the incidence of cardiovascular disease, the ultimate goal of treatment should be to achieve target control of glucose, BP, and lipids [133].

## Conclusions

The mechanism of hypertension in T2DM is complex, involving multiple neuro-humoral and metabolic factors, and both conditions share several similar aspects of pathophysiology. For type 2 diabetic patients with coronary artery disease, the optimal BP targets (especially for diastolic BP) vary based on clinical status in a given individual, and the strategy of BP control should be individualized to minimize adverse events and maximize benefits. Novel information as such is useful for clinicians and researchers to further add new knowledge on pathophysiology and therapeutic goal in diabetes and hypertension.

## Data Availability

Not applicable. No new datasets were generated for this manuscript.

## Abbreviations

ACC: American College of Cardiology
ACEI: angiotensin-converting enzyme inhibitor
AGE: advanced glycation end products
AHA: American Heart Association
ARB: angiotensin receptor blocker
BP: blood pressure
CFI: collateral flow index
CTO: chronic total occlusion
CTRP: C1q tumor necrosis factor related protein
DDP4: dipeptidyl peptidase 4
eNOS: endothelial nitric oxide synthase
esRAGE: endogenous secretory RAGE
GLP-1: glucagon-like peptide 1
HDL: high-density lipoprotein
NO: nitric oxide
oxLDL: oxidized low-density lipoprotein
PCDA: predominant collateral donor artery
PCI: percutaneous coronary intervention
PON: peroxidase
RAAS: renin–angiotensin–aldosterone system
RAGE: receptor for advanced glycation end products
ROS: reactive oxide spices
SGLT2: sodium-glucose cotransporter 2
sRAGE: soluble RAGE
